# Delirium after cardiac arrest: incidence, risk factors, and association with neurologic outcome—insights from the Freiburg Delirium Registry

**DOI:** 10.1007/s00392-024-02575-3

**Published:** 2024-11-18

**Authors:** Dawid Leander Staudacher, Laura Heine, Alexander Maier, Klaus Kaier, Adrian Heidenreich, Jonathan Rilinger, Felix Arne Rottmann, Paul Marc Biever, Alexander Supady, Tobias Wengenmayer, Dirk Westermann, Markus Jäckel

**Affiliations:** 1 Department of Medicine III (Interdisciplinary Medical Intensive Care), Medical Center, University of Freiburg, Faculty of Medicine, University of Freiburg, Freiburg, Germany; 2 Department of Diagnostic and Interventional Radiology, Medical Center-Faculty of Medicine, University of Freiburg, Freiburg, Germany; 3 Department of Cardiology and Angiology, Heart Center Freiburg University, Faculty of Medicine, University of Freiburg, Hugstetter Strasse 55, 79106 Freiburg, Germany; 4 Department of Cardiology and Angiology, Faculty of Medicine, Center of Big Data Analysis in Cardiology (CeBAC), Heart Center Freiburg University, University of Freiburg, Freiburg, Germany; 5 Faculty of Medicine and Medical Center, Institute of Medical Biometry and Statistics, University of Freiburg, Freiburg, Germany; 6 Department of Nephrology, University Hospital Freiburg, Faculty of Medicine, University of Freiburg, Freiburg, Germany

**Keywords:** Cardiac arrest, Resuscitation, Delirium, Neurologic outcome

## Abstract

**Aim:**

Delirium in patients treated in the intensive care unit (ICU) is linked to adverse outcome, according to previous observations. However, data on patients recovering after cardiac arrest are sparse. The aim of this study was to assess incidence, risk factors, and outcome of patients with delirium after cardiac arrest in the Freiburg Delirium Registry (FDR).

**Methods:**

In this retrospective registry study, all patients after cardiac arrest treated in the Freiburg University Medical Center medical ICU between 08/2016 and 03/2021 were included. Delirium was diagnosed using the Nursing Delirium screening scale (NuDesc), assessed three times daily. Favorable neurological outcome was defined as cerebral performance category (CPC) score at ICU discharge ≤ 2.

**Results:**

Two hundred seventeen patients were included and among them, delirium was detected in one hundred ninety-nine (91.7%) patients. Age was independently associated with the incidence of delirium (*p* = 0.003), and inversely associated with the number of delirium-free days (*p* < 0.001). Favorable neurological outcome was present in 145/199 (72.9%) with, and 17/18 (94.4%) patients without delirium (*p* = 0.048). While the incidence of delirium was not independently associated with a favorable neurologic outcome, the number of delirium-free days strongly predicted the primary endpoint [OR 2.14 (1.73–2.64), *p* > 0.001].

**Conclusion:**

Delirium complicated the ICU course in almost all patients after cardiac arrest. The number of delirium-free days was associated with favorable outcome while incidence of delirium itself was not.

**Graphical abstract:**

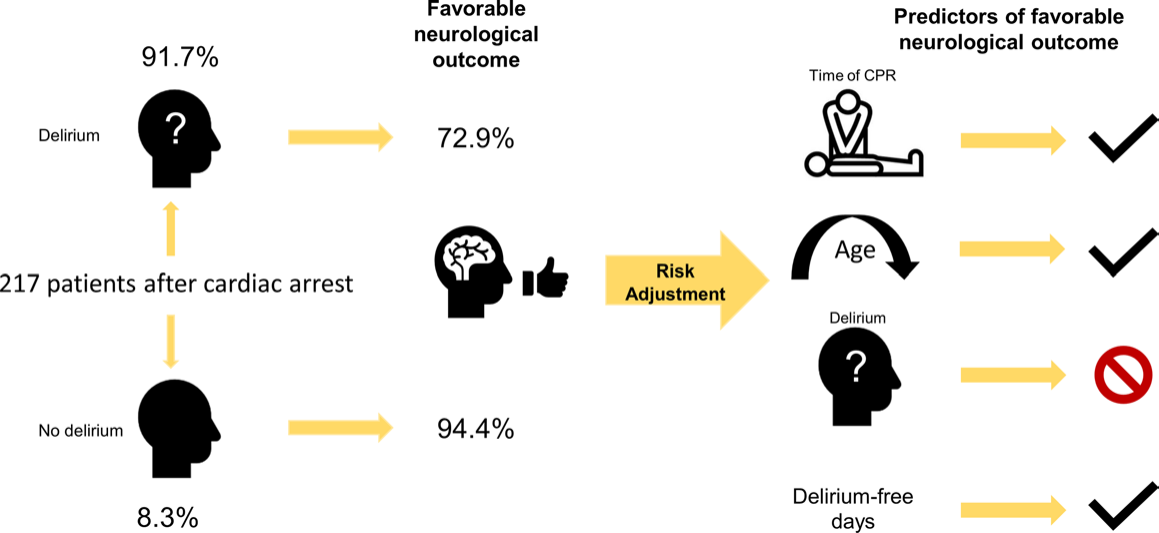

**Supplementary Information:**

The online version contains supplementary material available at 10.1007/s00392-024-02575-3.

## Introduction

Delirium, also known as “acute encephalopathy,” is a common complication of patients treated in the intensive care unit (ICU). According to a recent review and meta-analysis, the incidence was as high as 31% in a mixed ICU cohort [1]. However, incidence of delirium varies widely depending on the investigated patient cohort and the assessment methods used [2, 3]. Diagnosis of delirium is important for early intervention, as delirium incidence as well as duration are associated with adverse outcome including longer hospital stays, morbidity, and mortality [4–9].

Although delirium after cardiac arrest has been first described as early as 1967 described as organic brain syndrome, data on delirium are still limited in the context of post-cardiac arrest ICU treatment [10]. As the presence of anoxic brain injury complicates the diagnosis of delirium, post-cardiac arrest patients were frequently excluded from delirium studies [7]. In patients admitted to ICUs after cardiac arrest, post-cardiac arrest brain injury is the main reason of mortality and long-term disability [11]. An association of “short-term” brain encephalopathy, defined as delirium, and “long-term” brain encephalopathy, defined as hypoxic brain dysfunction, is currently under debate.

The primary aim of this study was to assess the incidence of delirium in patients following cardiac arrest. Secondary objectives included identifying risk factors for delirium in this patient cohort and analyzing the potential association with favorable neurological outcome.

## Methods

We conducted an investigator-initiated single-center retrospective cohort study. All patients from the Freiburg Delirium Registry (FDR) treated from August 2016 until March 2021 were included in our analysis. All patients > 18 years of age with cardiac arrest and either cardiopulmonary resuscitation (CPR) for ≥ 5 min, or a Glasgow Coma Scale (GCS) of ≤ 7 after return of spontaneous circulation (ROSC), were included. Exclusion criteria comprised discharge or death before extubation, as delirium assessment would not be feasible in these cases, and cannulation for extracorporeal membrane oxygenation (ECMO), given their presumed high likelihood of multifactorial delirium. Patients with severe hypothermia were also excluded, as post-CPR neurological pathology differs markedly from that of normothermic cardiac arrest [12].

The data analysis was conducted in a blinded manner, with patient identities concealed, and was conducted under ethics approval from the Ethics Committee of Albert Ludwigs University of Freiburg (file number 387/19). All scientific methods were carried out in accordance with relevant guidelines, regulations, the STROBE guideline for case–control studies, and the Declaration of Helsinki. Given that only retrospective data were included in the study, informed consent was waived by the ethics committee.

### Patient selection and data collection

All outcome variables were evaluated by manual case-by-case review of medical and patient records. Since only data from the index hospital stay were evaluated, no patients were lost to a follow-up. Registry was checked for data integrity and plausibility according to the RECORD recommendations for data clearing [13].

### Local policy on treatment of patients after cardiac arrest

Patients after cardiac arrest [in-hospital (IHCA) as well as out-of-hospital (OHCA)] and CPR for ≥ 5 min, or CPR < 5 min and GCS ≤ 7, underwent a target temperature management (TTM). Typically, TTM was maintained for 24 h at 33 °C, followed by slow rewarming at a rate of 0.2 °C/h, and ensuring fever avoidance for 48 h. Early detection and treatment of the cause for cardiac arrest was advocated. Most patients underwent coronary angiography, computed tomography (CT), or both after cardiac arrest. The management of vasopressors and fluid therapy was based on individual patient needs and clinical assessment of the intensivist in charge. Target mean arterial pressure (MAP) was > 80 mmHg for the first 24 h, and > 65 mmHg afterward till 08/2019. Since 08/2019, target MAP was > 65 mmHg [14]. A lung-protective ventilation was advocated targeting paCO_2_ 35–45 mmHg and paO_2_ ≥ 70 mmHg. For analgosedation, sufentanil and isoflurane or propofol were typically used targeting RASS-4 during the first 24 h after arrest. After reaching normothermia subsequent to TTM, an immediate wake-up trial was advocated.

### Definition of delirium and outcome

Delirium was defined by Nursing Delirium screening scale (NuDesc) ≥ 2 in at least one assessment and in selected patients confirmed by the documented assessment in the electronic files. The NuDesc score is routinely assessed by specially trained nurses in all ICU patients at least once per 8-h shift, corresponding to three assessments daily. To minimize a short-term observation bias, nurses perform a “representative screening” that reflects the patient’s overall condition during their shift. If a representative screening cannot be documented due to the patient’s fluctuating condition, multiple screenings may be performed. The NuDesc is approved, easy to use, and has high sensitivity and specificity for the detection of delirium [15–17]. In selected cases with conflicting results from NuDesc and the documented delirium assessment, a retrospective adjudication was performed. The motoric subtype of delirium was defined according to literature using the Richmond agitation and sedation scale (RASS), which is assessed at least three times daily on our ICU [18]. Specifically, hyperactive delirium was presumed when delirium was diagnosed and RASS was ≥ 1 in at least two consecutive evaluations [19]. Hypoactive delirium was presumed when RASS was ≤ 0 in at least two consecutive evaluations. Mixed delirium was presumed in case of alternant positive and negative RASS evaluations.

Delirium-free days within a 10-day period [referred to as delirium-free days (10)] were defined as days with a NuDesc score ≤ 1 within the first 10 days after the initial documented delirium evaluation, which typically occurred shortly after extubation. For patients discharged from the ICU without delirium before day 10, all subsequent days following discharge were considered delirium-free. Conversely, for patients who were reintubated or deceased before day 10, all days following reintubation or death were considered non-delirium-free. Neurologic outcome was determined by cerebral performance category (CPC) score at ICU discharge. Favorable neurological outcome was defined as CPC ≤ 2 [20].

### Local standard for delirium management

In patients with suspected or diagnosed delirium, our local protocol recommends a combination of pharmacological and non-pharmacological interventions. Non-pharmacological measures include reducing or discontinuing sedatives, ensuring adequate pain management, promoting daytime activation, reorientation by staff, involving relatives, and optimizing the patient’s environment (e.g., adequate daylight, quiet rooms with fewer patients). Other steps include quiet alarm management, minimal monitoring, removal of unnecessary cannulas, and encouraging oral feeding to restore the day-night cycle. Pharmacological treatments are only considered if these measures prove insufficient. Risperidone is the first-line treatment, with haloperidol as the second line, both administered at low doses and discontinued as soon as delirium resolves or improves.

### Statistical methods

All relevant data are given in standardized tables, either as n (%) for categorical data or as median and interquartile range (25th–75th) for continuous data.

For data analysis, SPSS (version 26, IBM Statistics) and Prism (version 10, GraphPad) were employed. For statistical analysis, Mann–Whitney *U* test was used for analysis of continuous variables. For categorical variables, Fisher’s exact test was used when number of expected values was smaller than five, otherwise Pearson’s Chi-squared test was performed. Delirium-free days were compared using the Mann–Whitney *U* test. Risk factors for delirium and delirium-free days were tested by multivariable regression analysis. Predictors for delirium were predefined according to literature heaving a plausible effect on delirium incidence. Similarly, predictors of outcome were predefined and tested in a multivariable regression analysis. Odds ratio (OR) with 95% confidence interval (CI) are reported as computed by the regression analysis or Fisher’s exact test. A *p* value of < 0.05 was considered statistically significant. Youden’s *J* was calculated as sensitivity + specificity − 1. The value of *J* was determined from the receiver operating characteristic (ROC) curve, where it represents the maximum sum of sensitivity and specificity.

## Results

### Study population

**Fig. 1:**
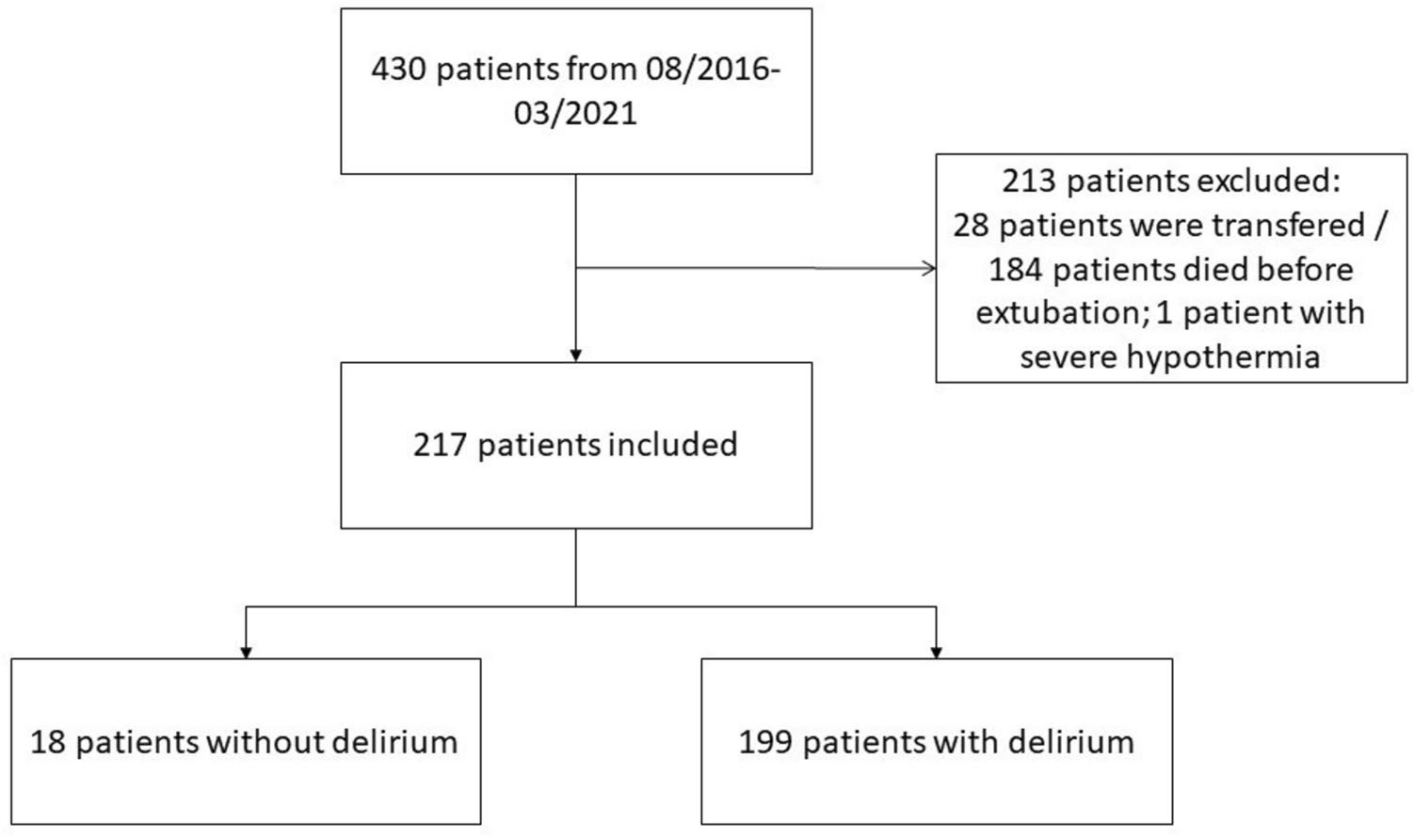
Flowchart indicating number of included and excluded patients. Data are given as number of patients

In the FDR, 430 patients after cardiac arrest were registered. Of these, 213/430 (49.5%) patients were excluded according to predefined exclusion criteria. Specifically, 28/430 (6.5%) due to a transferal before delirium evaluation, 184/430 (42.8%) due to death before delirium evaluation, and 1/430 (0.2%) due to profound hypothermic arrest. This led to 217/430 (50.5%) patients included in the analysis (Fig. 1). Mean age was 63 (55–72) years and 51/217 (23.5%) were female.

### Delirium incidence, presentation, and risk factors

**Fig. 2:**
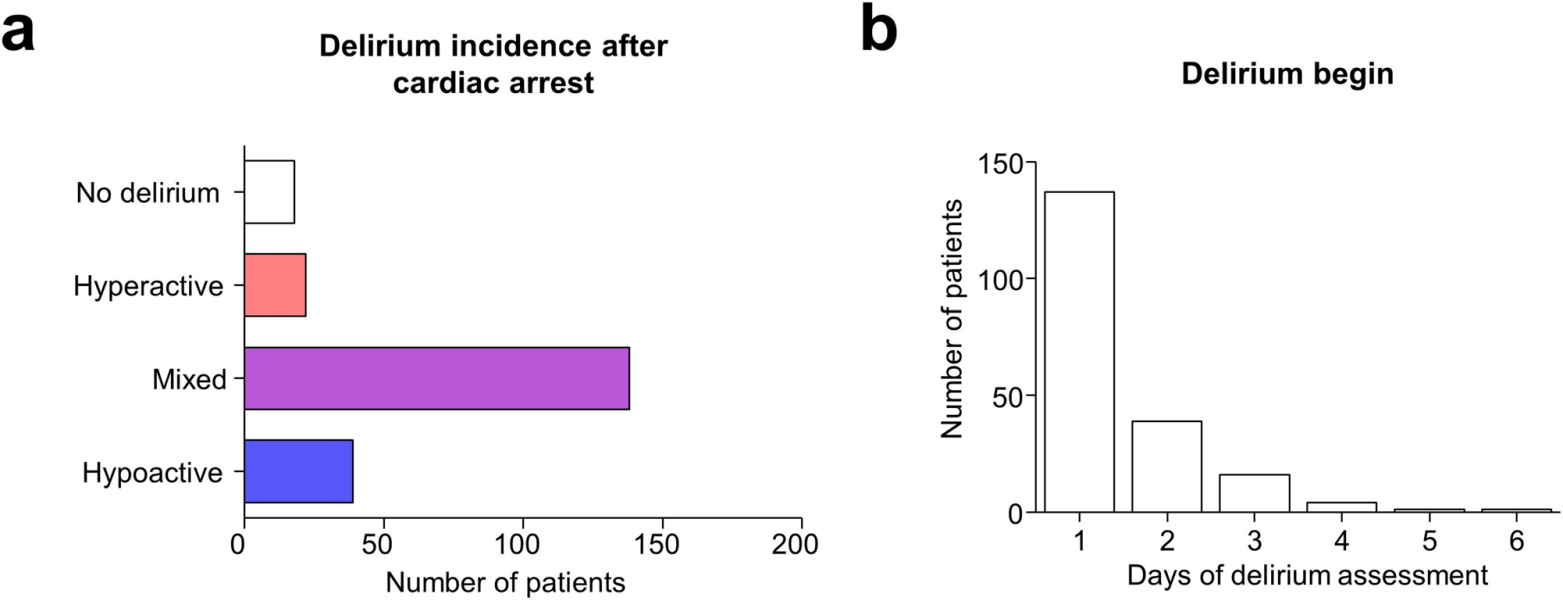
Delirium incidence (**a**) and begin (**b**) in patients after cardiac arrest

Delirium was diagnosed in 199/217 (91.7%) of all patients. Mixed delirium was the most common form of delirium, detected in 138/199 (69.3%) patients, followed by hypoactive 39/199 (19.6%) and hyperactive delirium 22/199 (11.1%). The median delirium-free days (10) were 6 (1–8) in patients with delirium. In 88.4% of all patients with delirium, delirium began during the first 2 days after extubation (Fig. 2).

Patients with delirium were older compared to patients without [63 (56–73) versus 51 (39–64) years; *p* = 0.001] and had more often arterial hypertension, while other comorbidities including neurologic and psychiatric diseases were similar. For baseline characteristics, see Table 1. Duration of ICU stay was significantly longer in patients with delirium compared to patients without and cause for cardiac arrest was more often of cardiac origin. No significant differences were identified concerning bystander CPR, shockable initial rhythm, no-flow duration, and low-flow duration. Duration of invasive ventilation was significantly longer in patients with delirium compared to patients without, as was the duration from cardiac arrest to first spontaneous breathing. No differences were identified between the use of isoflurane and propofol as sedatives (Table 2). No difference was identified concerning dosage of sedatives at various time points and delirium (supplemental Fig. 1).

**Table 1:** Baseline characteristics of all patients

	No delirium (*N* = 18)	Delirium (*N* = 199)	*p* value
Age	51 (39–64)	63 (56–73)	**0.001**
Female	5 (27.8%)	46 (23.1%)	0.771
Comorbidities			
Coronary heart disease	3 (16.7%)	46 (23.1%)	0.769
Arterial hypertension	4 (22.2%)	93 (46.7%)	**0.045**
Pulmonary disease	3 (10.0%)	42 (21.1%)	0.134
Liver disease	0 (0%)	14 (7.0%)	0.613
Chronic kidney disease	1 (5.6%)	29 (14.6%)	0.479
Peripheral/cerebral arterial occlusive disease	1 (5.6%)	13 (6.5%)	1.000
Neurological disease	1 (5.6%)	23 (11.6%)	0.700
Psychiatric disease/dementia	1 (5.6%)	25 (12.6%)	0.703
Alcohol abuse	2 (11.1%)	20 (10.1%)	0.701
Drug abuse	0 (0%)	7 (3.5%)	1.000

*p* value reported in bold if difference is significant (*p* < 0.05). Data are given as median and interquartile range (25th–75th) or number of patients (percent of all patients in group

**Table 2:** Clinical characteristics of all patients

	No delirium (*N* = 18)	Delirium (*N* = 199)	*p* value
ICU stay (days)	5.1 (4.3–6.8)	8.4 (6.0–12.6)	** < 0.001**
Delirium-free days (10)	10 (10–10)	6 (1–8)	** < 0.001**
Mortality	1 (5.6%)	24 (12.1%)	0.701
CPC at ICU discharge	1 (1–2)	1 (1–3)	0.084
Favorable neurological outcome	17 (94.4%)	145 (72.9%)	0.048
TISS 10	15 (12–19)	15 (10–19)	0.947
SAPS 2	39 (32–52)	46 (38–55)	0.057
Primary sedative Isoflurane	13 (72.2%)	165 (82.9%)	0.331
Primary sedative Propofol	5 (27.8%)	34 (17.1%)	0.331
Cardiac cause of arrest	9 (50.0%)	147 (73.9%)	**0.031**
OHCA	12 (66.7%)	164 (82.4%)	0.117
Witnessed cardiac arrest	16 (88.9%)	175 (87.9%)	1.000
Bystander CPR	12 (66.7%)	96 (48.2%)	0.134
Shockable initial rhythm	10 (55.6%)	126 (63.3%)	0.514
No-flow duration (min)	0 (0–2)	0 (0–5)	0.271
Duration of CPR (min)	12 (9–16)	15 (10–23)	0.157
Days of invasive ventilation	2.3 (1.9–3.5)	3.1 (2.3–4.8)	**0.009**
Time of first spontaneous breathing (days)	1.7 (1.5–1.9)	2.0 (1.8–2.4)	**0.002**
Renal replacement therapy	1 (5.6%)	21 (10.6%)	1.000

*p* value reported in bold if difference is significant (*p* < 0.05). Data are given as median and interquartile range (25th–75th) or number of patients (percent of all patients in group)

**Fig. 3:**
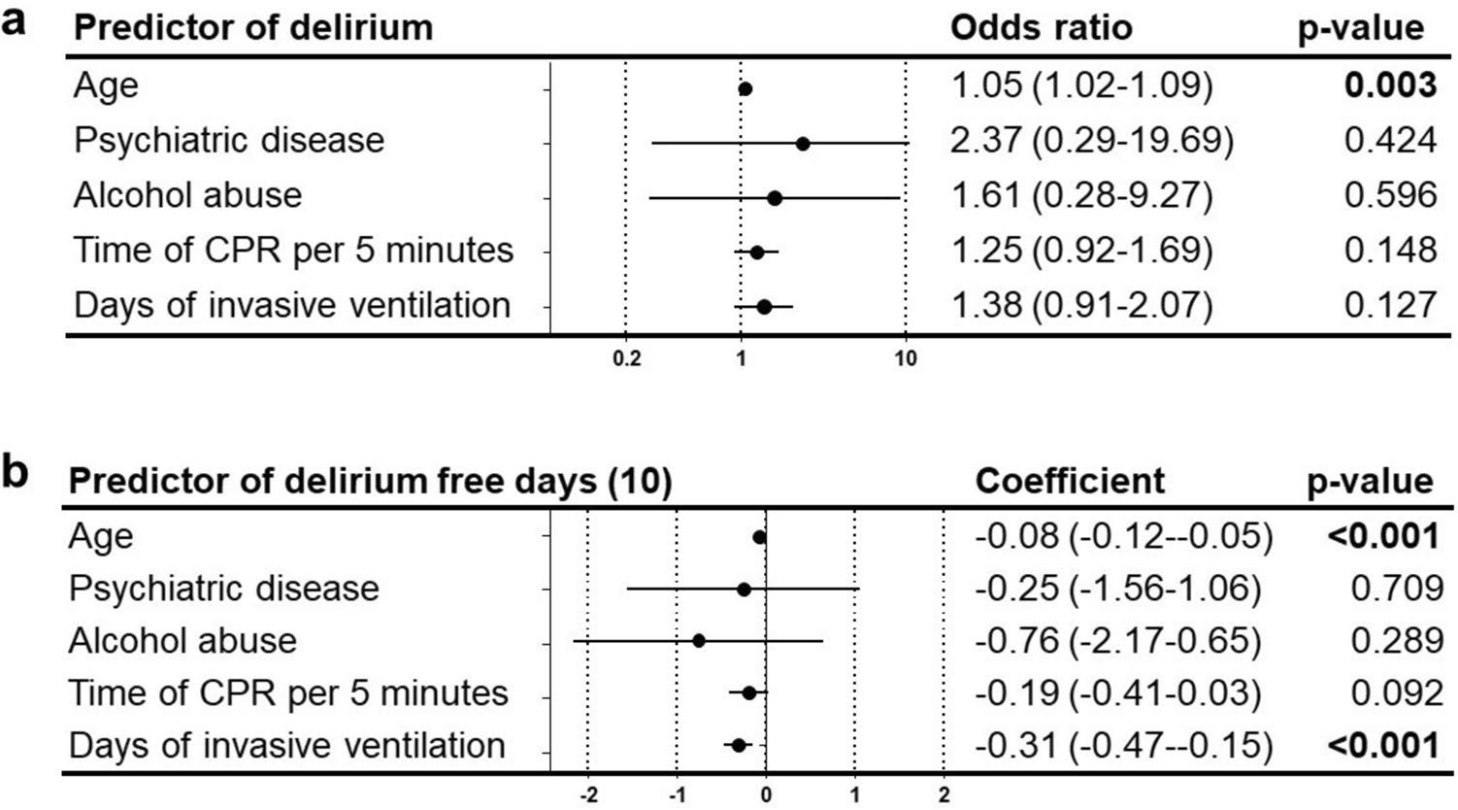
Predictors for delirium and delirium-free days (10). Multivariable logistic regression analysis with odds ratio (95% confidence interval) of predictors for delirium. Odds ratios > 1 mark positive predictors, odds ratios < 1 negative predictors (**a**). Multivariable linear regression analysis with regression coefficients of different predictors for delirium-free days 10 days after extubation. Coefficients > 0 mark predictors of more delirium free days, coefficients < 0 mark predictors of less delirium free days (**b**)

Only age was significantly associated with incidence of delirium with an OR 1.05 (1.02–1.09) in our dataset. Age and the duration of invasive ventilation were significantly associated with the number of delirium-free days (Fig. 3).

### Delirium and neurologic outcome

**Fig. 4:**
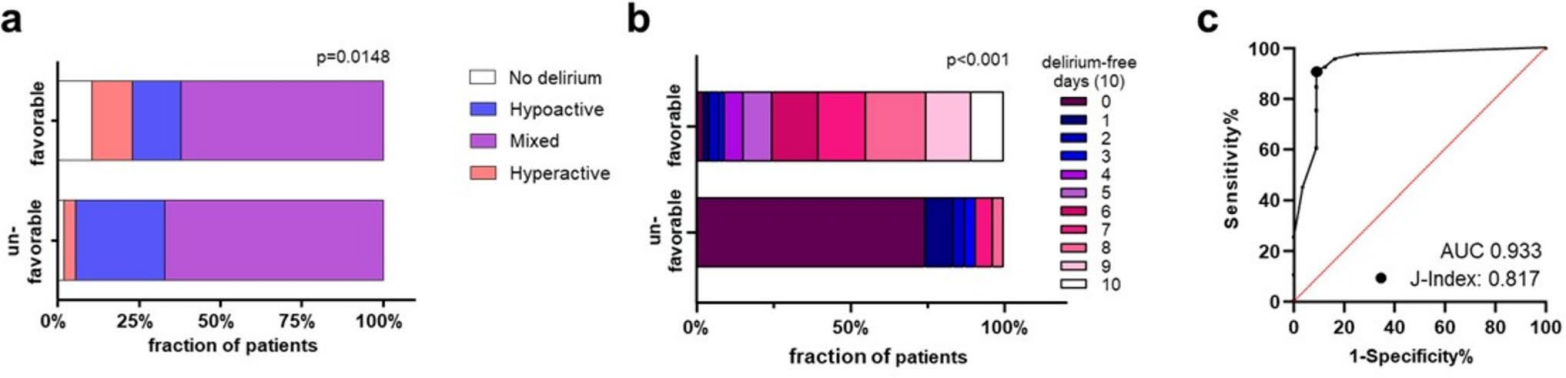
Delirium and outcome. Delirium presentation and outcome (**a**), delirium-free days analyzed 10 days after extubation and outcome (**b**), ROC for favorable neurological outcome and delirium-free days (10) (**c**)

A favorable neurological outcome was documented in 162/217 (74.7%) patients. Outcome in patients without delirium was significantly more often favorable compared to patients with delirium (94.4% versus 72.9%; *p* = 0.048) and hyperactive delirium was significantly more often present in patients with favorable outcome (Fig. 4a).

Patients with favorable neurological outcome had significantly more delirium-free days (10) [7 (6.0–8.75) versus 0 (0–0.5), *p* < 0.001] (Fig. 4b). In the receiver operating characteristic (ROC), delirium-free days (10) predicted the neurological outcome with an area under ROC of 0.933 (*p* < 0.001) and the best discriminator at > 3.5 delirium-free days (Youdens *J* 0.817, likelihood-ratio 9.81) (Fig. 4c).

**Fig. 5:**
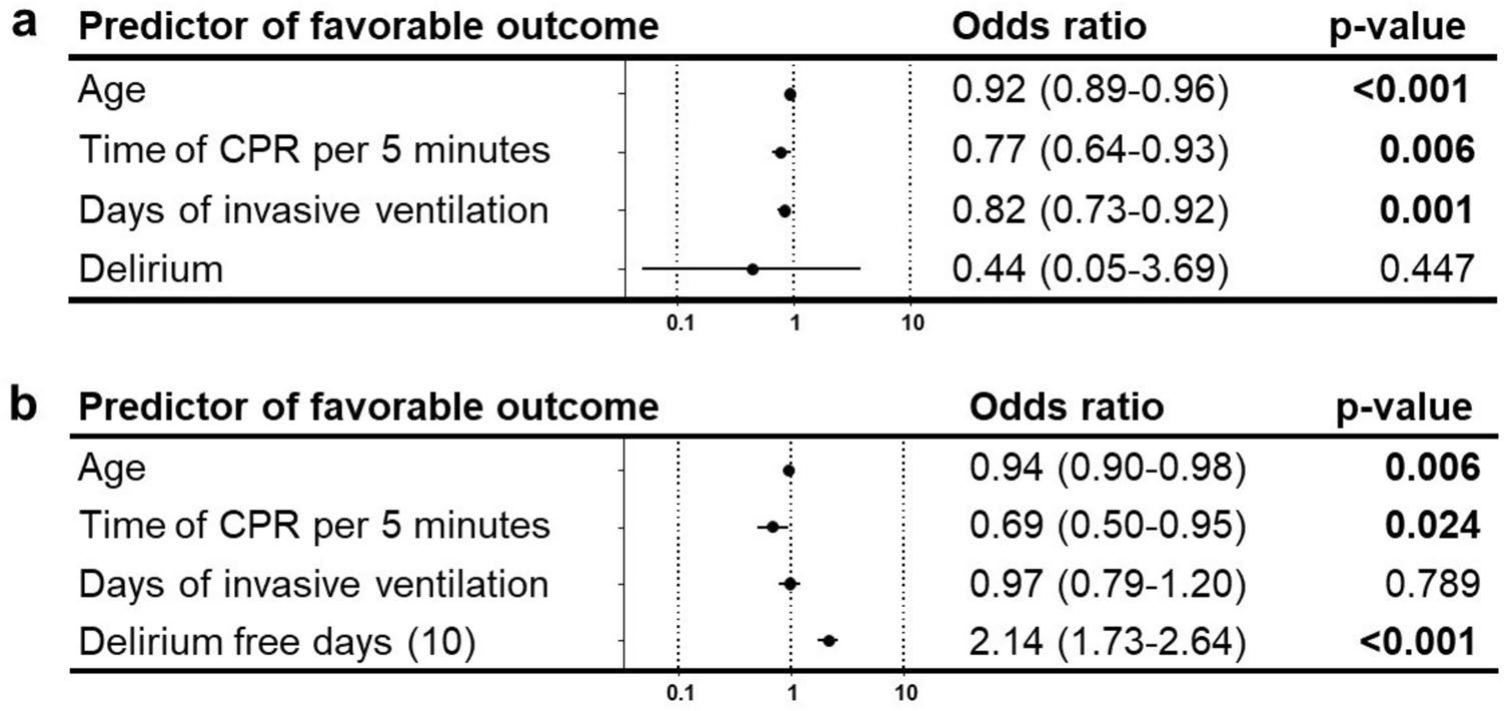
Predictors of favorable outcome. Multivariable logistic regression analysis with odds ratio (95% confidence interval) for predictors of favorable outcome including delirium (**a**) and delirium-free days 10 days after extubation (**b**). Odds ratios > 1 mark positive predictors, odds ratios < 1 negative predictors

While the presence of delirium was not associated with favorable outcome, the number of delirium-free days (10) was a good predictor of favorable neurologic outcome (Fig. 5).

## Discussion

Incidence of delirium after cardiac arrest in the FDR was 91.7%.

This is significantly higher than recently published incidence of 31% in a systematic meta-analysis comprising over 27,000 ICU patients [1]. Importantly, only view data exist on patients after cardiac arrest and only one study focused on these patients. Pollock et al. reported a 100% incidence of delirium in survivors of cardiac arrest treated with mild therapeutic hypothermia in a small retrospective analysis of 107 patients [21]. Our data support this finding pointing out a very high incidence of delirium after cardiac arrest compared to the general ICU population. The presentation of delirium after cardiac arrest with mixed delirium being the most common seen in our cohort, however, is well in line with data from mixed ICU cohorts [22].

Due to the retrospective nature of data presented, we can only speculate on reasons for the very high incidence of delirium after cardiac arrest. We have shown recently that cardiac arrest is a risk factor of delirium in a cohort of patients with acute myocardial infarction [23]. Therefore, cerebral low flow in the context of cardiac arrest or the post-resuscitation treatment might trigger delirium. Potential candidates are the hypoxic brain injury, deep sedation during targeted temperature management, metabolic dysregulation, and high doses of sedatives. The ongoing STEPCARE study (NCT05564754), which is currently randomizing over 3500 patients to 3 different interventions, including TTM- and sedation-free post-resuscitation care, promises to enhance our understanding of this crucial complexity.

This study excluded roughly half of the patients in whom delirium could not be screened, primarily because they died before extubation. Therefore, the results presented are based on an all-comers registry of patients who survived CPR until extubation. We cannot exclude the possibility that with advancements in post-CPR care, more patients may survive until extubation, potentially altering the incidence of delirium compared to the patients included in this study.

Age was the only significant predictor of delirium in our study in accordance to literature [3, 7]. Interestingly, other known risk factors for delirium including psychiatric diseases and alcohol abuse were not associated with the incidence of delirium in our cohort [24–26]. Likewise, duration of CPR and invasive ventilation were not associated with delirium. The most likely explanation might be the omnipresence of delirium being overshadowed by the predominant trigger of cardiac arrest and the post-resuscitation care. However, the fact that almost all patients were diagnosed with delirium limits the evaluation of risk factors that could be predictive of delirium. Whether other potential risk factors such as CPR duration, shockable initial rhythm, no-flow and low-flow duration are predictors for delirium should be reevaluated in a larger cohort.

Even though patients without delirium more frequently exhibited a favorable neurological outcome, 73% of patients with delirium also achieved a favorable neurological outcome. In our multivariable analysis, we were able to show that age, time of CPR, and necessity of prolonged invasive ventilation seem to be clearly more important predictors of an unfavorable neurologic outcome than delirium. Consequently, a young patient with short duration of CPR and invasive ventilation should still be expected to have a favorable neurological outcome, even when having delirium.

According to our data, delirium appears to be a universal complication after cardiac arrest. Interestingly however, the probability of a favorable neurologic outcome attenuated with increasing duration of delirium.

### Limitations

When discussing the results presented in our study, some limitations have to be considered. We present single-center retrospective data. Therefore, our results should be considered hypotheses-generating only and have to be confirmed in larger trials. In addition, we do not have follow-up data of the patients discharged. As we defined the neurologic outcome at the time of ICU discharge, the CPC may still have changed during rehabilitation, especially in patients with an unfavorable outcome. Since all patients included in this study received TTM, the data presented may not be generalizable to patients who underwent CPR without TTM. In addition, there are data indicating that the specificity of other screening tests such as the CAM-ICU may be higher than the NuDesc used in our registry [17]. However, no validation of delirium screening scores for patients after cardiac arrest exists. The NuDesc assesses five different symptoms including “psychomotoric retardation.” In particular, this assessment point cannot differentiate between patients with delirium or hypoxic brain damage. Consequently, a possible missclassification of patients with hypoxic brain injury cannot be excluded. However, this highlights the complexity of diagnosing delirium in post-cardiac arrest patients, where overlapping symptoms of brain injury and delirium complicate clinical interpretation. Furthermore, this potential overlap makes it difficult to determine causality. Whether delirium per se worsens the neurological outcome or is the expression of hypoxic brain damage, which we rather assume, remains unclear and requires further research.

Since we did not use structured clinical interviews, some variables are likely to be underreported.

## Conclusion

Delirium is a very frequent complication [incidence (91.7%)] after cardiac arrest but not necessary associated with an unfavorable neurologic outcome. Since the duration of delirium was associated with unfavorable outcome, more research is needed, to determine if delirium is a valid therapeutic target or a surrogate for excessive brain injury.

## Supplementary Information

Below is the link to the electronic supplementary material.

Supplementary file1 Supplemental figure 1 Mean dosage of Sufentanyl, Noradrenalin, primary sedative Isoflurane (MAC; N=13/165) and primary sedative Propofol (N=5/34) in all patients determined 6, 12, 24, 48, 72 and 120 hours after CPR. Significance is calculated by 2way ANOVA (JPG 231 KB)

## Data Availability

The datasets used and analyzed during the current study are available from the corresponding author on reasonable request.

## References

[1] Krewulak KD, Stelfox HT, Leigh JP, Ely EW, Fiest KM (2018) Incidence and prevalence of delirium subtypes in an adult ICU: a systematic review and meta-analysis. Crit Care Med 46:2029–2035. 10.1097/CCM.0000000000003402 30234569 10.1097/CCM.0000000000003402

[2] Vasilevskis EE, Han JH, Hughes CG, Ely EW (2012) Epidemiology and risk factors for delirium across hospital settings. Best Pract Res Clin Anaesthesiol 26:277–287. 10.1016/j.bpa.2012.07.003 23040281 10.1016/j.bpa.2012.07.003 PMC3580997

[3] Jäckel M, Aicher N, Bemtgen X, Rilinger J, Zotzmann V, Biever PM, Supady A, Stachon P, Duerschmied D, Wengenmayer T, Bode C, Staudacher DL (2021) Advantages of score-based delirium detection compared to a clinical delirium assessment-a retrospective, monocentric cohort study. PLoS ONE 16:e0259841. 10.1371/journal.pone.0259841 34843524 10.1371/journal.pone.0259841 PMC8629257

[4] Ely EW, Shintani A, Truman B, Speroff T, Gordon SM, Harrell FE, Inouye SK, Bernard GR, Dittus RS (2004) Delirium as a predictor of mortality in mechanically ventilated patients in the intensive care unit. JAMA 291:1753–1762. 10.1001/jama.291.14.1753 15082703 10.1001/jama.291.14.1753

[5] Pisani MA, Kong SYJ, Kasl SV, Murphy TE, Araujo KLB, van Ness PH (2009) Days of delirium are associated with 1-year mortality in an older intensive care unit population. Am J Respir Crit Care Med 180:1092–1097. 10.1164/rccm.200904-0537OC 19745202 10.1164/rccm.200904-0537OC PMC2784414

[6] Pauley E, Lishmanov A, Schumann S, Gala GJ, van Diepen S, Katz JN (2015) Delirium is a robust predictor of morbidity and mortality among critically ill patients treated in the cardiac intensive care unit. Am Heart J 170(79–86):86.e1. 10.1016/j.ahj.2015.04.013 26093867 10.1016/j.ahj.2015.04.013

[7] Boncyk CS, Rengel KF, Pandharipande PP, Hughes CG (2019) In the ICU—delirium post cardiac arrest. Curr Opin Crit Care 25:218–225. 10.1097/MCC.0000000000000615 30985357 10.1097/MCC.0000000000000615 PMC6902119

[8] Pandharipande PP, Girard TD, Jackson JC, Morandi A, Thompson JL, Pun BT, Brummel NE, Hughes CG, Vasilevskis EE, Shintani AK, Moons KG, Geevarghese SK, Canonico A, Hopkins RO, Bernard GR, Dittus RS, Ely EW (2013) Long-term cognitive impairment after critical illness. N Engl J Med 369:1306–1316. 10.1056/NEJMoa1301372 24088092 10.1056/NEJMoa1301372 PMC3922401

[9] Jäckel M, Aicher N, Rilinger J, Bemtgen X, Widmeier E, Wengenmayer T, Duerschmied D, Biever PM, Stachon P, Bode C, Staudacher DL (2021) Incidence and predictors of delirium on the intensive care unit in patients with acute kidney injury, insight from a retrospective registry. Sci Rep 11:17260. 10.1038/s41598-021-96839-x 34446816 10.1038/s41598-021-96839-x PMC8390667

[10] Druss RG, Kornfeld DS (1967) The survivors of cardiac arrest. A psychiatric study. JAMA 201:291–296 6071722

[11] Sandroni C, Cronberg T, Sekhon M (2021) Brain injury after cardiac arrest: pathophysiology, treatment, and prognosis. Intensive Care Med. 10.1007/s00134-021-06548-2 34705079 10.1007/s00134-021-06548-2 PMC8548866

[12] Lott C, Truhlář A, Alfonzo A, Barelli A, González-Salvado V, Hinkelbein J, Nolan JP, Paal P, Perkins GD, Thies K-C, Yeung J, Zideman DA, Soar J (2021) European resuscitation council guidelines 2021: cardiac arrest in special circumstances. Resuscitation 161:152–219. 10.1016/j.resuscitation.2021.02.011 33773826 10.1016/j.resuscitation.2021.02.011

[13] Benchimol EI, Smeeth L, Guttmann A, Harron K, Hemkens LG, Moher D, Petersen I, Sørensen HT, von Elm E, Langan SM (2016) Das RECORD-Statement zum Berichten von Beobachtungsstudien, die routinemäßig gesammelte Gesundheitsdaten verwenden. Z Evid Fortbild Qual Gesundhwes 115–116:33–48. 10.1016/j.zefq.2016.07.010 27837958 10.1016/j.zefq.2016.07.010 PMC5330542

[14] Jakkula P, Pettilä V, Skrifvars MB, Hästbacka J, Loisa P, Tiainen M, Wilkman E, Toppila J, Koskue T, Bendel S, Birkelund T, Laru-Sompa R, Valkonen M, Reinikainen M (2018) Targeting low-normal or high-normal mean arterial pressure after cardiac arrest and resuscitation: a randomised pilot trial. Intensive Care Med 44:2091–2101. 10.1007/s00134-018-5446-8 30443729 10.1007/s00134-018-5446-8 PMC6280836

[15] Gaudreau J-D, Gagnon P, Harel F, Tremblay A, Roy M-A (2005) Fast, systematic, and continuous delirium assessment in hospitalized patients: the nursing delirium screening scale. J Pain Symptom Manag 29:368–375. 10.1016/j.jpainsymman.2004.07.009 10.1016/j.jpainsymman.2004.07.009 15857740

[16] Bergjan M, Zilezinski M, Schwalbach T, Franke C, Erdur H, Audebert HJ, Hauß A (2020) Validation of two nurse-based screening tools for delirium in elderly patients in general medical wards. BMC Nurs 19:72. 10.1186/s12912-020-00464-4 32760215 10.1186/s12912-020-00464-4 PMC7393733

[17] Luetz A, Heymann A, Radtke FM, Chenitir C, Neuhaus U, Nachtigall I, von Dossow V, Marz S, Eggers V, Heinz A, Wernecke KD, Spies CD (2010) Different assessment tools for intensive care unit delirium: which score to use? Crit Care Med 38:409–418. 10.1097/CCM.0b013e3181cabb42 20029345 10.1097/CCM.0b013e3181cabb42

[18] Sessler CN, Gosnell MS, Grap MJ, Brophy GM, O’Neal PV, Keane KA, Tesoro EP, Elswick RK (2002) The Richmond Agitation-Sedation Scale: validity and reliability in adult intensive care unit patients. Am J Respir Crit Care Med 166:1338–1344. 10.1164/rccm.2107138 12421743 10.1164/rccm.2107138

[19] Peterson JF, Pun BT, Dittus RS, Thomason JWW, Jackson JC, Shintani AK, Ely EW (2006) Delirium and its motoric subtypes: a study of 614 critically ill patients. J Am Geriatr Soc 54:479–484. 10.1111/j.1532-5415.2005.00621.x 16551316 10.1111/j.1532-5415.2005.00621.x

[20] Gravesteijn BY, Schluep M, Disli M, Garkhail P, Dos Reis MD, Stolker R-J, Endeman H, Hoeks SE (2020) Neurological outcome after extracorporeal cardiopulmonary resuscitation for in-hospital cardiac arrest: a systematic review and meta-analysis. Crit Care 24:505. 10.1186/s13054-020-03201-0 32807207 10.1186/s13054-020-03201-0 PMC7430015

[21] Pollock JS, Hollenbeck RD, Wang L, Holmes B, Young MN, Peters M, Ely EW, McPherson JA, Vasilevskis EE (2016) Delirium in survivors of cardiac arrest treated with mild therapeutic hypothermia. Am J Crit Care 25:e81–e89. 10.4037/ajcc2016581 27369041 10.4037/ajcc2016581 PMC5240926

[22] Collet MO, Caballero J, Sonneville R, Bozza FA, Nydahl P, Schandl A, Wøien H, Citerio G, van den Boogaard M, Hästbacka J, Haenggi M, Colpaert K, Rose L, Barbateskovic M, Lange T, Jensen A, Krog MB, Egerod I, Nibro HL, Wetterslev J, Perner A (2018) Prevalence and risk factors related to haloperidol use for delirium in adult intensive care patients: the multinational AID-ICU inception cohort study. Intensive Care Med 44:1081–1089. 10.1007/s00134-018-5204-y 29767323 10.1007/s00134-018-5204-y

[23] Jäckel M, Zotzmann V, Wengenmayer T, Duerschmied D, Biever PM, Spieler D, von zurMühlen C, Stachon P, Bode C, Staudacher DL (2020) Incidence and predictors of delirium on the intensive care unit after acute myocardial infarction, insight from a retrospective registry. Catheter Cardiovasc Interv. 10.1002/ccd.29275 32926556 10.1002/ccd.29275

[24] Zhou Q, Zhou X, Zhang Y, Hou M, Tian X, Yang H, He F, Chen X, Liu T (2021) Predictors of postoperative delirium in elderly patients following total hip and knee arthroplasty: a systematic review and meta-analysis. BMC Musculoskelet Disord 22:945. 10.1186/s12891-021-04825-1 34772392 10.1186/s12891-021-04825-1 PMC8588632

[25] Horacek R, Krnacova B, Prasko J, Latalova K (2016) Delirium as a complication of the surgical intensive care. Neuropsychiatr Dis Treat 12:2425–2434. 10.2147/NDT.S115800 27703360 10.2147/NDT.S115800 PMC5036558

[26] Jäckel M, Aicher N, Biever PM, Heine L, Bemtgen X, Rilinger J, Zotzmann V, Supady A, Stachon P, Wengenmayer T, Bode C, Staudacher DL (2021) Delirium in critically ill patients with and without COVID-19—a retrospective analysis. JCM 10:4412. 10.3390/jcm10194412 34640428 10.3390/jcm10194412 PMC8509381

