# Supplementary figures and images for: Delirium after cardiac arrest: incidence, risk factors, and association with neurologic outcome—insights from the Freiburg Delirium Registry
Source: Clin Res Cardiol. 2024 Nov 18;114(10):1349–57. doi: 10.1007/s00392-024-02575-3 (PMC12460583; doi:10.1007/s00392-024-02575-3)

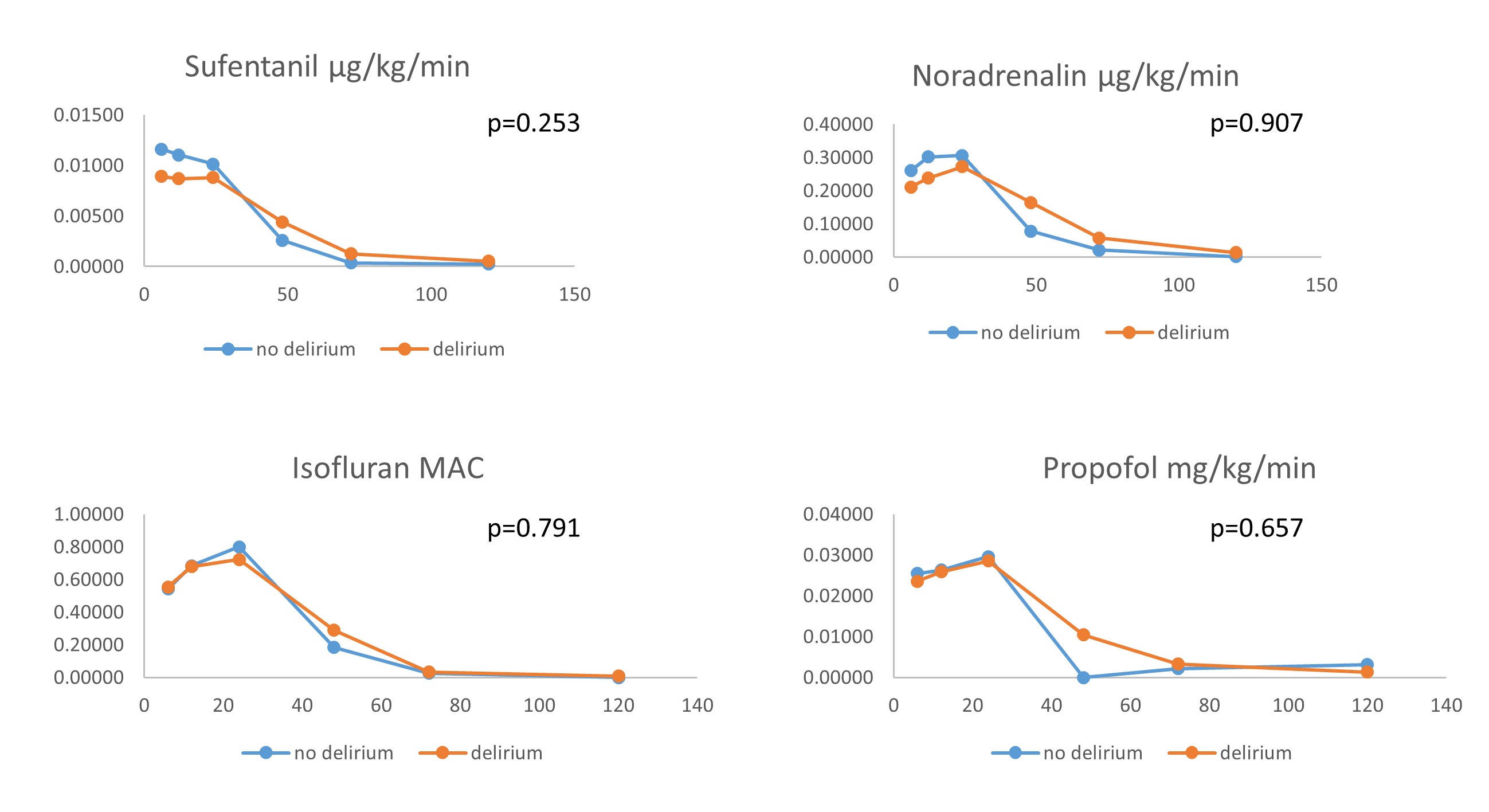

Supplement: Supplementary file 1 — Supplementary file1 Supplemental figure 1 Mean dosage of Sufentanyl, Noradrenalin, primary sedative Isoflurane (MAC; N=13/165) and primary sedative Propofol (N=5/34) in all patients determined 6, 12, 24, 48, 72 and 120 hours after CPR. Significance is calculated by 2way ANOVA (JPG 231 KB) [file 392_2024_2575_MOESM1_ESM.jpg]
